# Sphingosine-1-Phosphate (S-1P) Promotes Differentiation of Naive Macrophages and Enhances Protective Immunity Against *Mycobacterium tuberculosis*

**DOI:** 10.3389/fimmu.2019.03085

**Published:** 2020-01-24

**Authors:** Vinod Nadella, Lalita Sharma, Pankaj Kumar, Pushpa Gupta, Umesh D. Gupta, Srikant Tripathi, Suresh Pothani, S. S. Y. H. Qadri, Hridayesh Prakash

**Affiliations:** ^1^Laboratory of Translational Medicine, School of Life Science, University of Hyderabad, Hyderabad, India; ^2^Department of Experimental Animal Facility, National JALMA Institute for Leprosy and Other Mycobacterial Disease, Agra, India; ^3^Department of Bacteriology, National Institute of Research in Tuberculosis, Chennai, India; ^4^National Animal Resource Facility for Biomedical Research, National Institute of Nutrition, Indian Council of Medical Research Hyderabad, Hyderabad, India

**Keywords:** sphingolipids, tuberculosis, S-1P receptors, macrophage polarization, innate immunity, lungs, antimicrobial

## Abstract

Sphingosine-1-phosphate (S-1P) is a key sphingolipid involved in the pathobiology of various respiratory diseases. We have previously demonstrated the significance of S-1P in controlling non-pathogenic mycobacterial infection in macrophages, and here we demonstrate the therapeutic potential of S-1P against pathogenic *Mycobacterium tuberculosis* (H37Rv) in the mouse model of infection. Our study revealed that S-1P is involved in the expression of iNOS proteins in macrophages, their polarization toward M1 phenotype, and secretion of interferon (IFN)-γ during the course of infection. S-1P is also capable of enhancing infiltration of pulmonary CD11b+ macrophages and expression of S-1P receptor-3 (S-1PR3) in the lungs during the course of infection. We further revealed the influence of S-1P on major signaling components of inflammatory signaling pathways during *M. tuberculosis* infection, thus highlighting antimycobacterial potential of S-1P in animals. Our data suggest that enhancing S-1P levels by sphingolipid mimetic compounds/drugs can be used as an immunoadjuvant for boosting immunity against pathogenic mycobacteria.

## Introduction

Tuberculosis (TB) is a global disease and one of the major causes of mortality worldwide where an approximate one-third of the global population is infected with the causative agent *Mycobacterium tuberculosis* (1). Despite major advancements toward its treatment, several factors including increase in antibiotic-resistant *M. tuberculosis* strains (2), co-infections (3), and inadequate host–pathogen interactions (4) continue to pose major challenges to the health care system. Therefore, development of novel therapeutic approaches that could improve immunity against TB is a paramount requirement. During acute infection, alveolar macrophages acquire M1 phenotype (5, 6), secrete interferon (IFN)-γ, and mount Th1 response in the process of controlling infection in the lungs (7). In view of this, enrichment/stabilization of M1 phenotype represents one potential strategy for effective control of mycobacterial infection. Sphingolipids are active constituents of the mucus secreted by alveolar epithelium and protects the lung tissue from invading pathogens. Out of various sphingolipid metabolites, sphingosine-1-phosphate (S-1P), and ceramide are the best studied sphingolipids in the context of various respiratory pathologies (8–10). As S-1P and ceramide were known to exert opposite signaling in the host (11, 12), S-1P/ceramide rheostat would be a decisive parameter in predicting how cells would respond differentially to the same stimuli during disease progression. S-1P is a well-known secondary messenger that is pleiotropic in nature and orchestrates signaling mainly *via* G protein–coupled S-1P receptors 1–5 (13, 14). Several reports have suggested that temporal regulation of S-1P receptors may account for such pleiotropic effect of S-1P in a variety of cells (15, 16).

We have previously demonstrated that sphingosine kinase-1 (17), a critical enzyme of the sphingolipid metabolism, can control non-pathogenic mycobacterial infection in macrophages in an S-1P–dependent manner. On this note, we explored the role of S-1P in controlling pathogenic mycobacteria in the mouse model of infection, hypothesizing that enchasing S-1P levels may provide survival benefit to the host. In line with our hypothesis, this study reveals the S-1P and IFN-γ cross talk for the expression of iNOS proteins by macrophages, their polarization toward M1 phenotype, and augmenting pro-inflammatory immune responses. Our *M*. *tuberculosis* pulmonary challenge model demonstrated the potential of S-1P for enhancing the expression of iNOS proteins and their associated signaling proteins in augmenting pro-inflammatory immune response during the course of *M. tuberculosis* infection. Our data further demonstrated the upregulation of S-1PR3 and increased infiltration of CD11b+ alveolar myeloid cells (macrophages) in the lungs of *M. tuberculosis*–infected mice by S-1P during the course of infection.

Taken together, our data suggest that S-1P can control pathogenic mycobacteria and warrant the use of sphingolipids (S-1P or mimetic) as novel pharmacological approaches for augmenting immunity against mycobacterial infection.

## Results

### S-1P Skews M1 Polarization and Th1 Effector Response in Naive Macrophages

**Figure 1:**
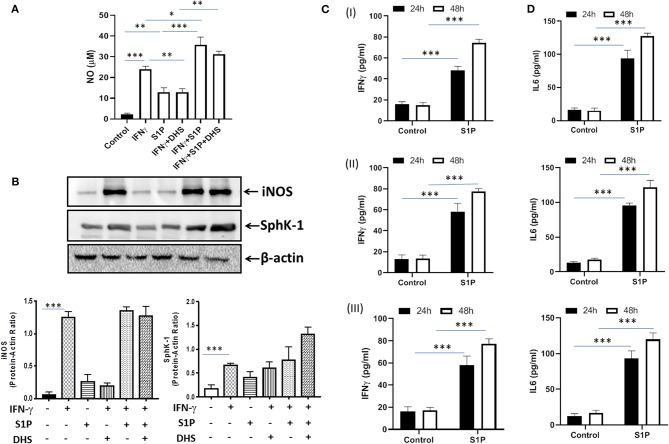
Sphingosine-1-phosphate (S-1P) skews M1 effector response in macrophages. **(A)** RAW264.7 macrophages were stimulated as indicated, and NO titers were quantified from their culture supernatants. Shown here is the μM of NO ± SEM from three independent experiments. **(B)** Whole cell lysates of macrophages used above were collected 24 h post-treatment culture and analyzed for the expression of iNOS **(M1 effector protein)** and Sphk-1 proteins by immune blotting. A representative blot from several repeats with similar outcome is shown. β-actin was used as a loading control. Mean density of each protein was quantified from various blots by ImageJ software, and the values were plotted in terms of relative protein expression. **(C)** RAW macrophages **(I)**, CD11b+ peritoneal **(II)**, and bone marrow–derived macrophages **(III)** were treated with S-1P for indicated time intervals. Cell culture supernatants were collected, and IFN-γ **(C)** and IL-6 **(D)** were measured by ELISA at 24 and 48 h, respectively. Shown here is the pg/ml of cytokines released during the course of infection ± SEM from three independent experiments. Statistical analyses were conducted using two-way ANOVA followed by Bonferroni posttest (*P < 0.05, **P < 0.01, ***P < 0.001).

Our previous study had demonstrated that S-1P/Sphk-1–mediated antimycobacterial responses are independent of tumor necrosis factor (TNF)-α (17); a component of immunity that is sufficient to eliminate a large number of intracellular pathogens. In view of this, we hypothesized that S-1P may enhance IFN-γ secretion that is capable of skewing M1 polarization of macrophages. To demonstrate this, macrophages were stimulated with IFN-γ and S-1P independently, and titers of inducible NO and expression of iNOS proteins were analyzed. In line with our hypothesis, S-1P and IFN-γ on their own induced NO titers (Figure 1A) and expression of iNOS proteins (Figure 1B) in macrophages. On these bases, we hypothesized the possible synergy between S-1P and IFN-γ for enhancing NO and iNOS levels in macrophages. Interestingly, co-stimulation of IFN-γ-treated macrophages with S-1P further enhanced the expression of NO (Figure 1A) and iNOS proteins (Figure 1B), providing the first evidence of S-1P/IFN-γ cross talk in M1 macrophages.

Sphk-1 catalyzes the production of S-1P, and inhibiting Sphk-1 enzymatic activity would inhibit the expression of iNOS in these macrophages. Interestingly, treatment of macrophages with dihydrospingosine (DHS) for inhibiting Sphk-1 activity resulted in inhibited IFN-γ-induced expression of iNOS proteins (Figure 1B), revealing a direct correlation of Sphk-1 proteins with IFN-γ-mediated M1 polarization of macrophages. On the basis of S-1P/IFN-γ-driven M1 polarization, we questioned whether S-1P on its own would skew pro-inflammatory immune response in naive macrophages. To test this, macrophages were treated with S-1P, and titers of IFN-γ (Figure 1C) and interleukin (IL)-6 (Figure 1D) were quantified in their culture supernatants at indicated time intervals. Following our hypothesis, S-1P enhanced the secretion of these cytokines by naive macrophages, revealing its adjuvant-like potential. These results revealed the involvement of S-1P in augmenting pro-inflammatory immune responses in macrophages, which are paramount for controlling *M. tuberculosis* infection.

### S-1P Promotes Protective Immune Response Against *M. tuberculosis*

**Figure 2:**
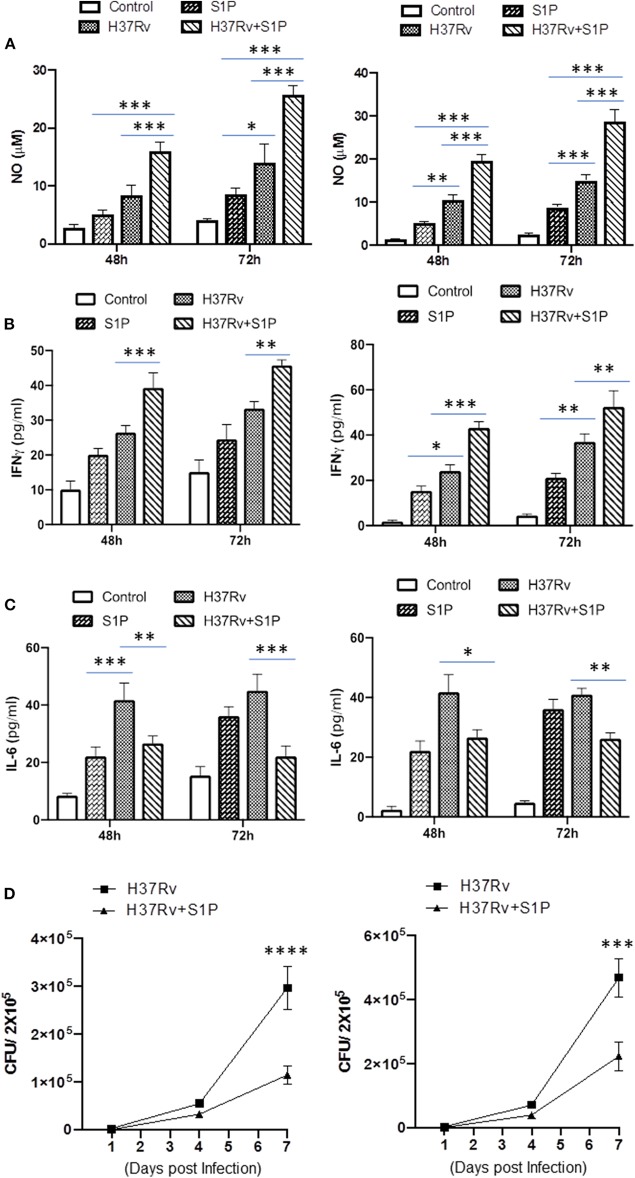
RAW 264.7 macrophages (Left Panel) and BMDMs (Right Panel) were infected with *M. tuberculosis* in presence of S-1P. Titres of NO **(A)**, IFN-γ **(B)**, and IL-6 **(C)** were analyzed in their culture supernatant. Shown here is the μM of NO ± SEM and pg/ml of cytokines ± SEM released during the course of infection from three independent experiments. Statistical analysis were conducted using two way Anova followed by Bonferroni post-test (*P < 0.05, **P < 0.01, ***P < 0.001, ****P < 0.0001). **(D)** Anti-mycobacterial influence of S-1P was also analyzed in same cells. Shown here CFU ± SEM from three independent experiments.

**Figure 3:**
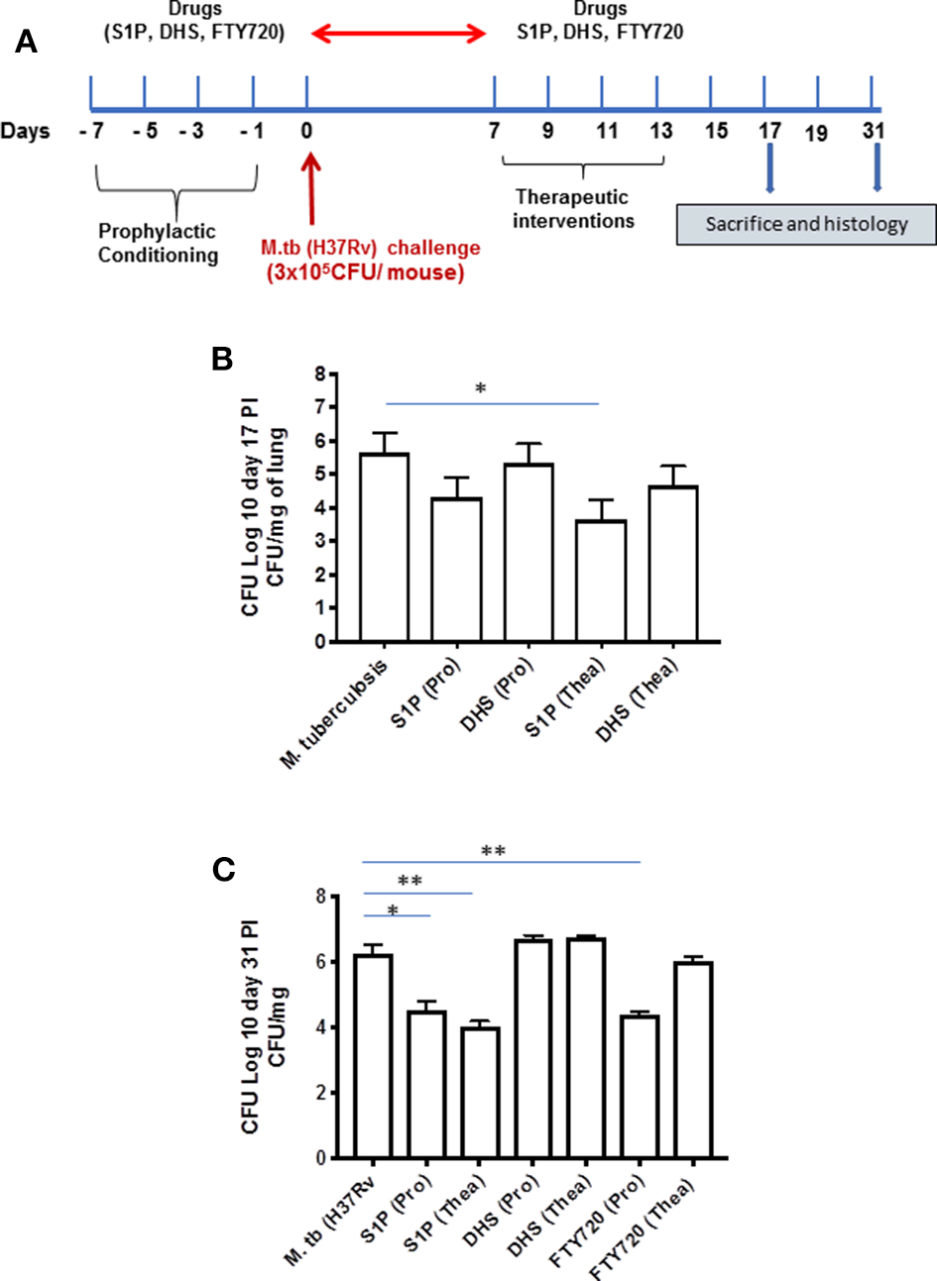
Sphingosine-1-phosphate (S-1P) controls *M. tuberculosis* burden and promotes Th1 effector immune response in infected macrophages. **(A)** Schematic representation of aerosol experiment detailing treatment schedules with respect to the *M. tuberculosis* challenge. **(B,C)** Mice from indicated groups were sacrificed on 17th and 31st postinfection day. Lung and spleen tissues were excised, and the number of bacteria was analyzed by plate-based method. Bacterial growth was monitored at 17th **(B)** and 31st **(C)** day postinfection in lung homogenates of mice infected with *M. tuberculosis* (H37Rv). Shown here is CFU ± SEM from three independent experiments. Statistical analysis were conducted using two way Anova followed by Bonferroni post-test (*P < 0.05, **P < 0.01).

**Figure 4:**
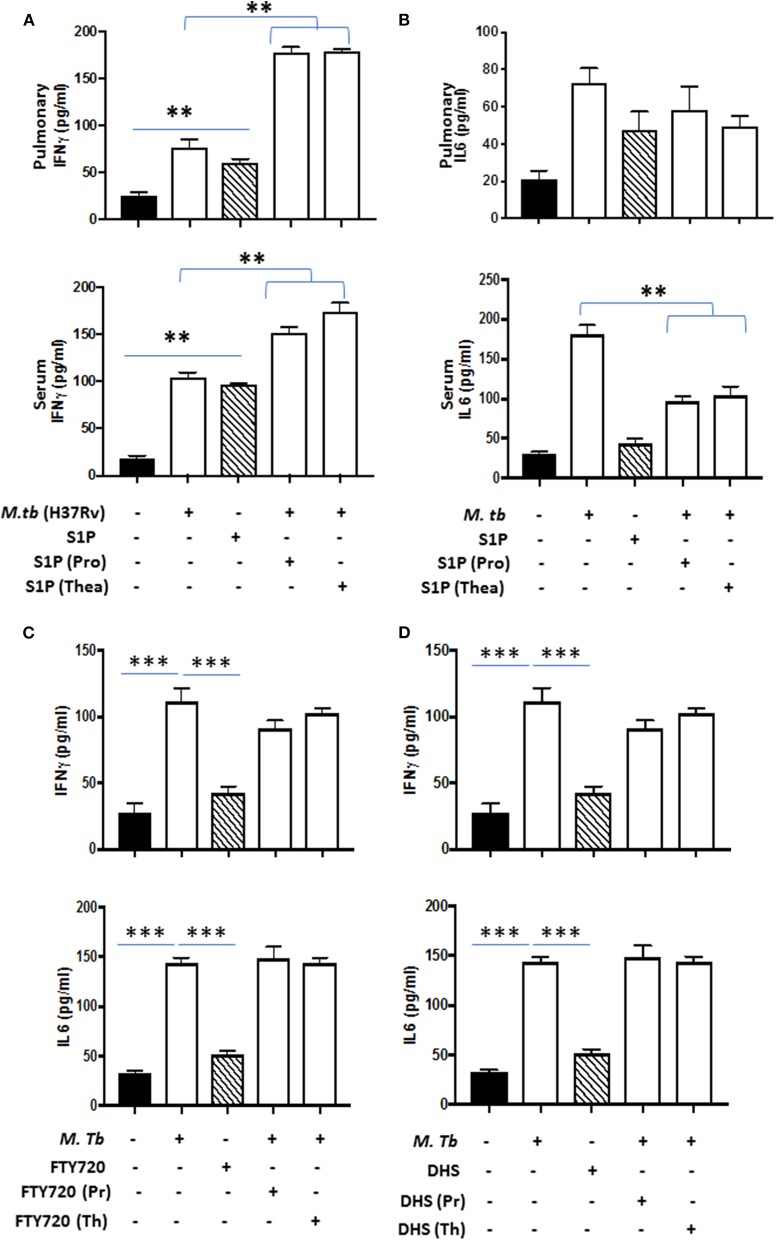
Lung homogenates and serum were prepared from S-1P conditioned mice and IFN-γ (**A**; both pulmonary compartment and serum) and IL-6 (**B**; both pulmonary compartment and serum) titres were quantified by ELISA. Similarly, IFN-γ and IL-6 titres were quantified from lungs homogenates of mice treated with FTY720 **(C)** and DHS **(D)** by ELISA. Shown here is the pg/ml of cytokines produced ± SEM from several mice used in each group. Statistical analysis were conducted using two-way Anova followed by Bonferroni post-test (**P < 0.01, ***P < 0.001).

**Figure 5:**
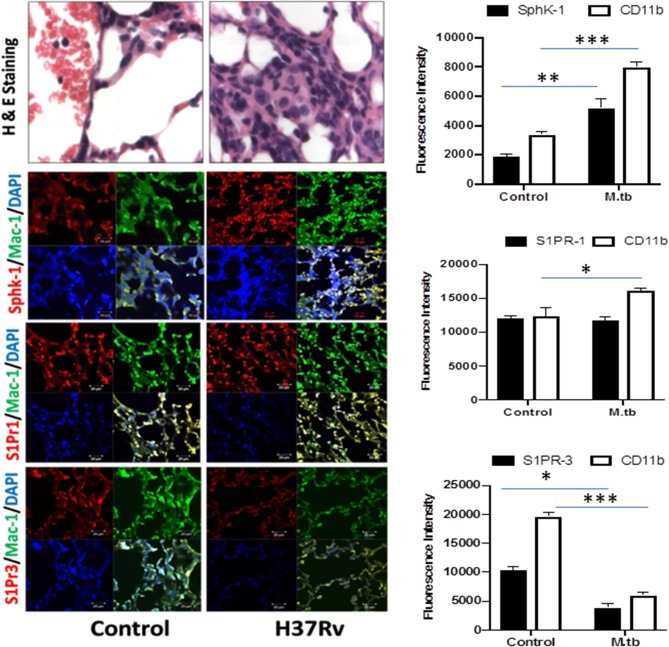
Increased infiltration of macrophages into the lung of *M. tuberculosis*–challenged animals. Sections of lungs from infected mice were analyzed for expression of sphingosine-1-phosphate receptor (S-1PR)1, S-1PR3, and SphK-1 and infiltration of CD11b+ macrophages by confocal microscopy. Shown here is the representative images of mouse lung from each group. Mean fluorescence intensities of Sphk-1, S-1PR1, and S-1PR3 expression were plotted against Mac1 expression. Statistical analyses were conducted using two-way ANOVA followed by Bonferroni posttest (*P < 0.05, **P < 0.01, ***P < 0.001).

**Figure 6:**
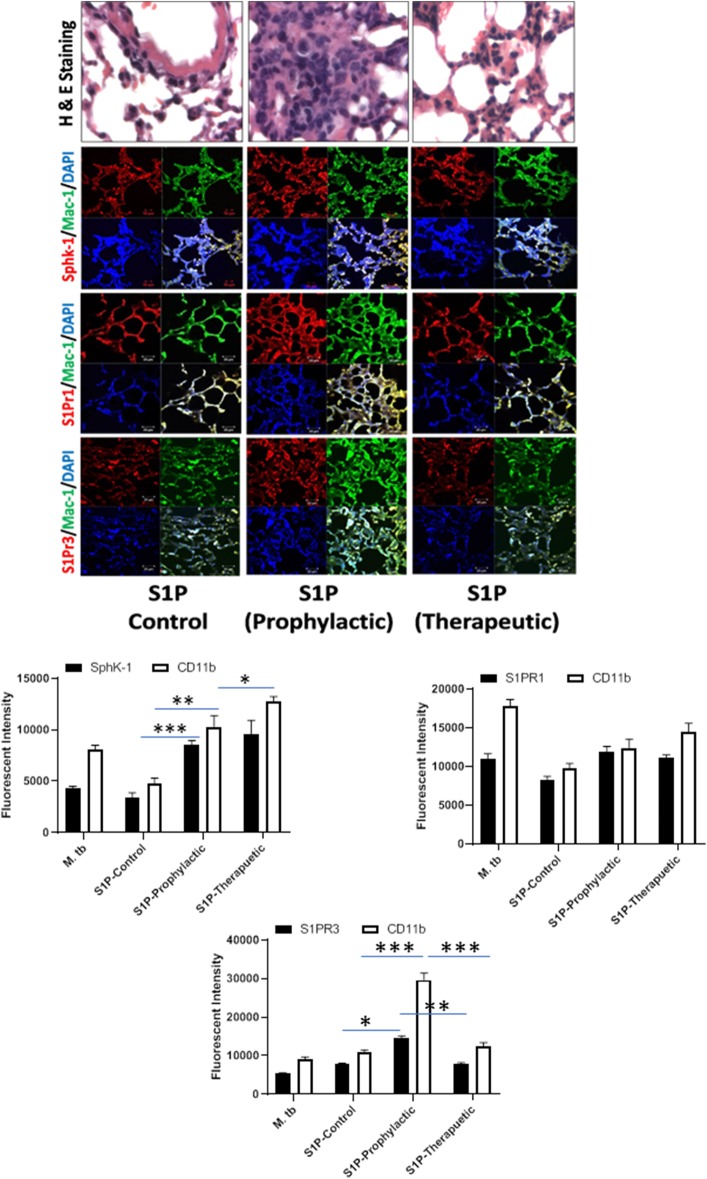
Sphingolipids modulate macrophage infiltration and pro-inflammatory response in *M. tuberculosis*–challenged animals. The infected mice were treated with sphingosine-1-phosphate (S-1P) as per Figure 3A, and cryosections of lung from these mice were analyzed for expression of S-1PR1, S-1PR3, and SphK-1 and infiltration of CD11b+ macrophages by confocal microscopy. Shown here is the representative images of mouse lung from each group. Mean fluorescence intensities of Sphk-1, S-1PR1, and S-1PR3 expression were plotted against Mac1 expression. Statistical analyses were conducted using two-way ANOVA followed by Bonferroni posttest (*P < 0.05, **P < 0.01, ***P < 0.001).

**Figure 7:**
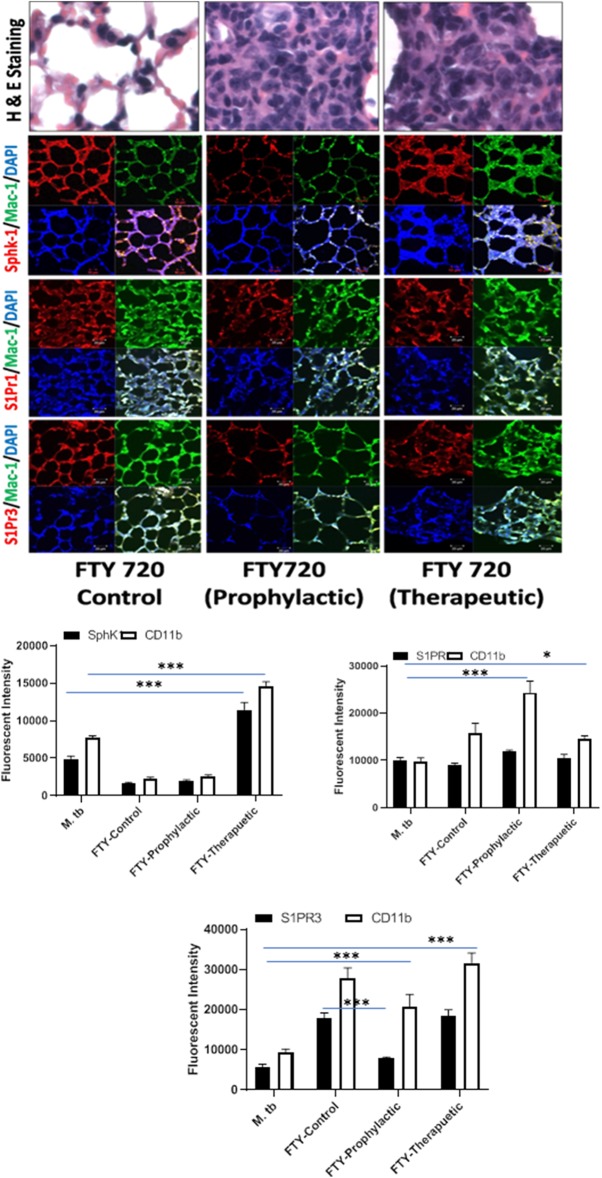
FTY-720 enhances sphingosine-1-phosphate receptor (S-1PR)3 in *M. tuberculosis*–challenged animals. The infected mice were treated with FTY-720 as per Figure 3A, and cryosections of lungs from these mice were analyzed for expression of S-1PR1, S-1PR3, and SphK-1 and infiltration of CD11b+ macrophages by confocal microscopy. Shown here is the representative images of mouse lung from each group. Mean fluorescence intensities of Sphk-1, S-1PR 1, and S-1PR3 expression were plotted against Mac1 expression. Statistical analyses were conducted using two-way ANOVA followed by Bonferroni posttest (*P < 0.05, ***P < 0.001).

**Figure 8:**
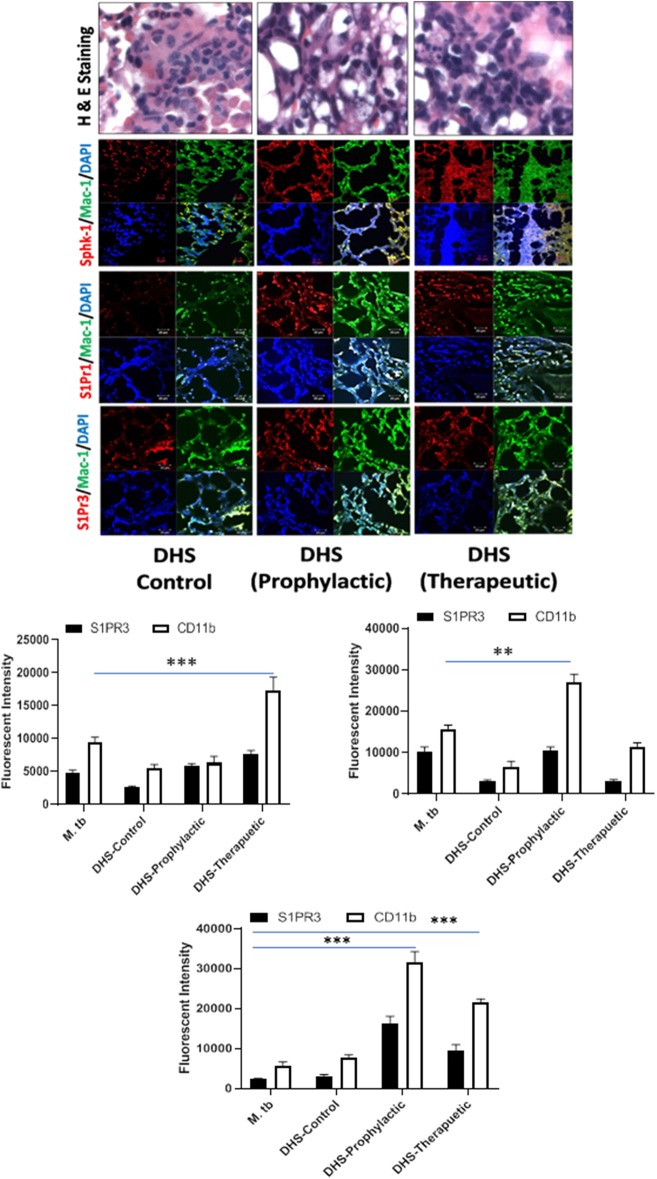
DHS failed to control *M. tuberculosis* burden due to reduced SphK-1 activity. The infected mice were treated with DHS as per Figure 3A, and cryosections of lungs from these mice were analyzed for expression of sphingosine-1-phosphate receptor (S-1PR)1, S-1PR3, and SphK-1 and infiltration of CD11b+ macrophages by confocal microscopy. Shown here is the representative images of mouse lung from each group. Mean fluorescence intensities of Sphk-1, S-1PR1, and S-1PR3 expression were plotted against Mac1 expression. Statistical analyses were conducted using two-way ANOVA followed by Bonferroni posttest (**P < 0.01, ***P < 0.001).

On the basis of S-1P/IFN-γ-driven M1 polarization, we argued whether S-1P would be able to skew Th1 immune response in macrophages against *M. tuberculosis* infection. To demonstrate this, RAW 264.7 macrophages (left panel; Figure 2) and bone marrow–derived macrophages (BMDMs; right panel; Figure 2) were infected with H37Rv, and pro-inflammatory immune responses were monitored *vis-à-vis* mycobacterial survival. Interestingly, treatment of infected macrophages with S-1P not only enhanced the generation of NO (Figure 2A) and secretion of IFN-γ (Figure 2B) over infected controls. Interestingly the same inhibited the secretion of IL-6 (Figure 2C) in the infected macrophage significantly and controlled mycobacterial survival in these macrophages (Figure 2D). On the basis of pro-inflammatory and antimycobacterial potential of S-1P *in vitro*, we anticipated for a similar impact of S-1P *in vivo*. To this end, a mouse model of *M. tuberculosis* pulmonary infection published by JALMA, Agra, India, was adopted, and the mice were infected with *M. tuberculosis* in the presence and absence of S-1P, FTY720 [to mitigate S-1P signaling (11, 14), and DHS to inhibit S1P production] (17) both in prophylactic as well as in therapeutic settings, respectively. Prophylactic conditioning of mice with various sphingolipid derivatives was done 1 week before infection. For that purpose, mice were injected with sphingolipid derivatives *via* intraperitoneal route, taking toxicity associated with intratracheal, and/or intravenous routes into consideration (18). Mice were injected with sphingolipid derivatives on every alternate day for a week and subsequently infected with *M. tuberculosis* (H37Rv) using an aerosol chamber (Inhalation Exposure System, Glas-Col Inc., IN, USA) (19). Thereafter, mice were kept for a week for the establishment of infection in their lungs. For analyzing the therapeutic potential of drugs, mice were treated with sphingolipid derivatives from seventh through 14th day postinfection (Figure 3A). Mice were sacrificed on the 17th and 31st day postinfection, and their lungs, spleen, and serum were excised aseptically for evaluating the bacterial growth, protein analysis, and histopathological analysis in the lung tissues. In accordance with our hypothesis, *M. tuberculosis* survival pattern analysis revealed that treatment of infected mice with S-1P (either prophylactically or therapeutically) controlled *M. tuberculosis* burden marginally by 17th day postinfection (Figure 3B) and significantly by 31st day postinfection (Figure 3C), demonstrating the anti-TB potential of S-1P. In line with our previous study, treatment of infected mice with DHS increased *M. tuberculosis* burden (Figure 3C). To substantiate the anti-TB potential of S-1P, the involvement of various S-1P receptors in controlling mycobacteria in S-1P–treated animals was analyzed. Mice were treated with FTY-720, expecting that FTY-720 would provide survival benefit to bacteria. Although treatment of mice with FTY-720 in prophylactic conditions controlled the infection significantly by 31st day postinfection (Figure 3C), the same could not influence the mycobacterial survival when applied therapeutically. Although these treatments altered the growth pattern of *M. tuberculosis* in lungs, the same could not influence the bacterial clearance during the course of infection (Supplementary Figure 1). On account of anti-TB potential of S-1P, we next analyzed the influence of S-1P on both pro-inflammatory response and innate immune signaling in infected lungs. In line with RAW and peritoneal macrophage responses, S-1P enhanced IFN-γ titers in lungs as well as in serum of infected mice (Figure 4A). Most intriguingly, S-1P inhibited IL-6 titers in the infected macrophages (Supplementary Figure 2) as well as in infected mice (Figure 4B), which is associated with mycobacterial burden and disease severity in TB patients. These observations not only revealed S-1P–mediated pro-inflammatory programming but also furnished a potential immune mechanism detailing how S-1P could control mycobacterial survival in these animals. In line with mycobacterial burden, both FTY-720 (Figure 4C) and DHS (Figure 4D) could not modify *M. tuberculosis*–induced titer of IFN-γ and IL-6 in the lung of infected mice. S-1P is chemotactic in nature and known to promote macrophage infiltration (20, 21). Therefore, we anticipated for an enhanced lung infiltration of CD11b+ macrophage population in S-1P–treated animals during the course of infection in mice. Histopathological analysis of *M. tuberculosis*–infected lungs revealed increased infiltration of CD11b+ pulmonary macrophages (Figure 5), which got further enhanced upon their treatment with either S-1P (Figure 6) or FTY-720 (Figure 7), respectively. Furthermore, both S-1P/FTY-720 enhanced the expression of Sphk-1 proteins (Figures 6, 7) in infected lungs, which would have accounted for enhanced expression of iNOS proteins in the infected lungs. Interestingly, both S-1P (Figure 6) and FTY-720 (Figure 7) enhanced the expression of S-1PR3, which is known to promote immune cell migration and antibacterial defenses against intracellular pathogens (16). Although DHS also enhanced the expression of S-1PR3 (Figure 8) and most surprisingly iNOS proteins as well (Supplementary Figure 3) in the lungs of DHS-treated animals, it could not control the mycobacterial burden.

**Figure 9:**
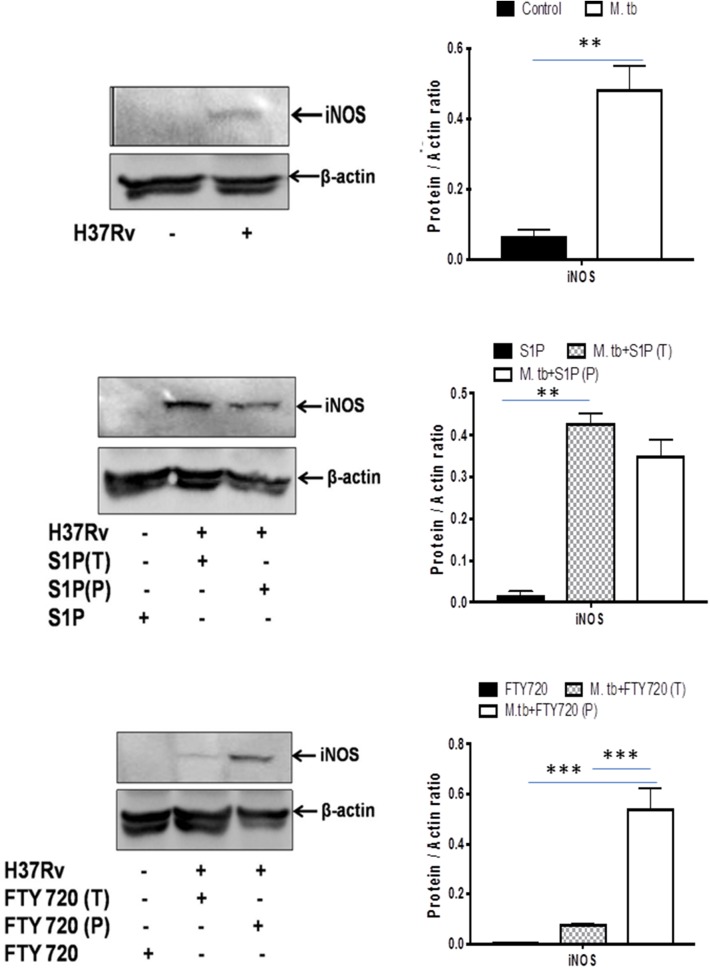
Sphingolipids promote M1 programming in the lung during the course of *M*. tuberculosis infection. *M. tubeculosis*–infected mice were conditioned with indicated sphingolipid drug derivatives, and whole lung lysates from these mice were purified and analyzed for M1 effector protein (iNOS) by Western blot. Shown here are the representative blots from each group. β-actin was used as a loading control. Densitometric analysis of the blot shown was quantified by ImageJ software, and the values were plotted in terms of relative protein expression. Statistical analyses were conducted using two-way ANOVA followed by Bonferroni posttest (**P < 0.01, ***P < 0.001).

On account of pro-inflammatory and antimycobacterial impact, we anticipated for M1 programming of macrophages in S-1P–treated animals during the course of infection. Indeed, in line with the above data, treatment of infected animals with S-1P enhanced the expression of iNOS proteins in the lung of the infected animals, which revealed M1 programming of S-1P (Figure 9) in the infected animals.

### S-1P Enhances Pro-inflammatory Signaling During *M. tuberculosis* Infection

**Figure 10:**
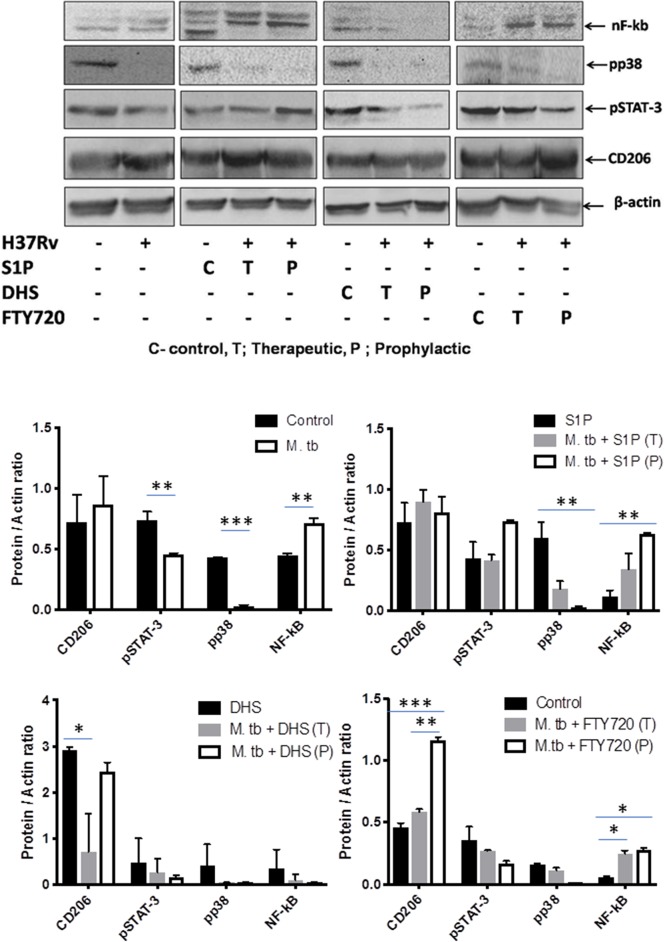
Sphingosine-1-phosphate (S-1P) promotes innate immune signaling during *M. tuberculosis* challenge. Whole lung lysates of infected mice treated with indicated sphingolipid drug derivatives were purified and analyzed for the expression of various key signaling proteins vis NF-κb, pp38, pSTAT-3, and CD206 by Western blot analysis. Shown are the representative Western blots obtained from each group. β-actin was used as a loading control. Densitometric quantification of the representative Western blots obtained from each group was analyzed by ImageJ, and the values were plotted in terms of relative protein expression. Statistical analyses were conducted using two-way ANOVA followed by Bonferroni posttest (*P < 0.05, **P < 0.01, ***P < 0.001).

On the basis of S-1P–mediated control of mycobacterial burden and concomitant pro-inflammatory/M1 programming in mice, we strongly anticipated for an enhanced innate immune signaling by S-1P in lungs. To address our hypothesis, the expressions of various signaling proteins that are known to be involved in translational activation of iNOS proteins in M1–polarized macrophages were analyzed. Interestingly, treatment with S-1P enhanced the expression of key signaling proteins, including phospho-MAPK (pp38), phospho-Nf-κB, and phospho-STAT3 in the lungs of infected animals during the course of the infection (Figure 10), which may account for S1P–mediated control of pathogenic mycobacteria in infected animals.

## Discussion

Sphingolipids are crucial bioactive molecules, and their therapeutic potential has been well-documented, especially in the context of respiratory tract infections, and acute lung injuries (22). Host pathogen interactions play a key role in determining the outcome of infection. In this context, our previous findings have suggested that sphingolipids (S-1P in particular) are important bio-macromolecules and serve as signaling components during mycobacterial infection in macrophages. Several reports have suggested that S-1P and ceramide rheostat and inter-se signaling leads to cystic fibrosis (CF), chronic abstractive pulmonary disorders (COPD), acute respiratory distress syndrome (ARDS), respiratory tract infections, and associated immunogenic inflammatory response (23, 24).

Sphingosine kinase 1 is one of the key enzymes of the sphingolipid pathways that phosphorylate sphingosine to S-1P. We have previously reported that sphingosine kinase-1 can control non-pathogenic mycobacterial infection in macrophages in an S-1P–dependent manner (17), and in the current study, we present its ability in controlling pathogenic mycobacteria in a mouse model system. The synergism between S-1P and IFN-γ for triggering M1 programming of infected macrophages accounted for mitigating mycobacterial burden, their dissemination, and the associated pathology. On these lines, we believe that S-1P can be used as an immune adjuvant for the management of mycobacterial diseases and warrant further investigation.

S-1P utilizes both extracellular and intracellular pathways for its biological responses. In the context of recruitment and inflammatory programming of immune cells, extracellular signaling *via* various S-1P receptors is decisive. Out of various receptors, S-1PR1 and S-1PR3 are important for immune cell recruitment and its activation. This led us to hypothesize their involvement in skewing anti-TB influence of S-1P and observed that treatment of infected mice with S-1P enhanced the expression of S-1PR3 against our expectation for S-1PR1. This might be that S-1P have utilized S-1PR3–associated signaling for restricting mycobacterial growth in mice (16) as shown in the context of other bacteria. This clearly reflects the compensation of S-1PR1 on S-1PR3, which is known to promote immune cell migration and antibacterial defenses against intracellular pathogens (16). Although FTY-720 also enhanced the expression of S-1PR3 in the lung of infected mice, it could not control *M. tuberculosis* burden in these mice, suggesting their possible signaling *via* other S-1P receptors offering survival benefit to bacteria. This is an interesting aspect of the study and warrant further analysis on temporal regulation of these receptors or associated signaling favoring immune escape of bacteria. Most interestingly, DHS also enhanced the expression of S-1PR3 and to our surprise iNOS proteins as well. However, DHS–mediated increase in S-1PR3 and iNOS expression could not control mycobacterial burden, which could be due to reduced Sphk-1 activity in these mice, thus highlighting the significance of intracellular signaling in the control of mycobacteria. Increased expression of S-1PR3 vis-à-vis pro-inflammatory micromilieu of S-1P–conditioned mice during the course of infection may account for one of the potential S-1P–directed antimycobacterial mechanisms in these animals. We believe that S-1PR3–positive macrophages in S-1P–treated mice, other than antibacterial responses on their own (25, 26), could have also promoted the lung infiltration of T cells for mounting an effective antimycobacterial response (27). Since S-1P is known to influence T and B cells during immunogenic inflammation, which is largely mediated by S-1P receptors (28, 29), we cannot exclude the role of T and B cells in S-1P–driven control of *M. tuberculosis* burden. It is likely that these cells, particularly CD8+ T cells could also have contributed directly or indirectly to the *in situ* M1 polarization and subsequent Th1 responses during the course of infection (28, 30). On the basis of the IFN-γ/IL-6 response, it is clear that S-1P may favor the host and improve the sensitivity of immune cells for IFN-γ-mediated antimycobacterial responses.

Taken together, our data potentially identify S-1P as one of the active constituents of metabolism having antimycobacterial efficacy. This study compelled us to believe that boosting sphingolipid levels (S-1P) in the host would offer therapeutic advantages in controlling mycobacterial infection. Thus, our study revealed the therapeutic efficacy of S-1P or mimitics for the management of tuberculosis (Supplementary Figure 4) and suggested that such pharmacological interventions may help both innate and adaptive immunity for the control of mycobacterial disease.

## Materials and Methods

### Antibodies and Reagents

All reagents were purchased from Sigma-Aldrich (UK), unless stated otherwise. RPMI 1640, penicillin streptomycin solutions were procured from Sigma-Aldrich. Recombinant mouse IFN-γ cytokine was purchased from eBiosciences (San Diego, CA). CD11b+ human and mouse MACS MicroBeads and LC Columns were purchased from Miltenyi Biotec. Primary antibodies including rabbit polyclonal iNOS, rabbit polyclonal CD-206, rabbit polyclonal, and mouse monoclonal β-actin were purchased from Santa Cruz Biotechnology. Rabbit monoclonal STAT3, pp38MAPK, pNF-kB, and Sphk-1 antibody were purchased from Santa Cruz. S-1PR1 and S-1PR3, HRP-linked anti-mouse IgG, and anti-rabbit IgG were purchased from Cell Signaling Technology. Anti-mouse/human-CD11b (CloneM1/70)-FITC-conjugated antibodies and their respective isotype control antibody including FITC rabbit IgG2bK (Clone RTK4530), PE Rat IgG2bK (Clone RTH4530), and PerCP/Cy5.5 rat IgG2bK (RTK4530) were purchased from Biolegend (Germany). Alexa fluor-488– and Alexa fluor 569–conjugated antibodies were purchased from Invitrogen. IFN-γ and IL-6 ELISA kits were purchased from R&D Systems (Darmstadt, Germany).

### Ethics Statement

All animal experiments were performed as per the guidelines laid down by Institutional Animal Ethical Committee under CPCSEA guidelines and approved by Institutional Animal Ethical Committee from University of Hyderabad (UH/IAEC/HP/2015/P19), Hyderabad, and JALMA, Agra.

### *M. tuberculosis* Infection of Mice

For animal infection, frozen aliquots of *M. tuberculosis* (H37Rv) were thawed, washed in phosphate buffer saline (PBS). For *in vivo* studies, C57BL6 mice purchased from NIN, Hyderabad, were maintained at 20–22°C and relative humidity of 50–70%. *M. tuberculosis* infection experiments were conducted at BSL-3 facility of JALMA, Agra, India. Six- to eight-week-old mice were kept in well-ventilated perplex boxes, and the mice were given standard diet of rodent pellets and water. Mice were treated with drugs (S-1P, DHS, FTY-720) 1 week before infection for prophylactic group and 1 week postinfection for therapeutic group. All drug treatments were done intraperitoneally and on alternate days as per schedule shown. Each mouse was challenged with *M. tuberculosis* (H37Rv) *via* respiratory route using an aerosol chamber (Inhalation Exposure System, Glas-Col Inc., IN, USA) for 45 min in 100 μl of saline. After infection, mice were kept in a controlled environment, and their health status was monitored regularly.

### Cell Culture

RAW 264.7 macrophages were purchased from ATCC and maintained in Roswell Park Memorial Institute medium (RPMI) containing amino acids and supplemented with 10% (v/v) fetal bovine serum (FBS) and 1% penicillin and streptomycin in CO_2_ incubator. To isolate CD11b+ peritoneal macrophages, C57BL/6j mice were injected with 1 ml of 4% brewer thioglycolate medium intraperitoneally, and peritoneal lavage was harvested at third day postinjection. Peritoneal lavage was centrifuged at 400 g for 8 min, and the cell pellet was resuspended in fresh serum-free RPMI. CD11b+ macrophages were purified by MACS-based separation method and were cultured in serum containing medium overnight. Macrophage monolayers were washed on the following day. For the preparation of BMDM, C57BL/6j mice were sacrificed by cervical dislocation, and both femurs were excised aseptically. Femurs were washed twice with ice-cold sterile PBS. Tibiae were cut from the femur at the joint and purged with ice-cold PBS using 5-ml syringes. The cell suspension were collected in 15-ml tubes and filtered through a 70-μm cell strainer to remove cellular debris. The cell suspensions were centrifuged at 400 g for 10 min at 4°C. RBCs in pellet were lysed by using ACK lysis buffer and removed by centrifugation. Cell pellets were dissolved in complete DMEM and incubated in the presence of GM-CSF for a week. To obtain BMDM, supernatants were discarded, and the attached macrophages were washed and detached by gentle pipetting the PBS across the dish. Cells collected were centrifuged at 1,200 rpm for 5 min and resuspended in 10 ml of BMDM cultivation media containing 10% fetal bovine serum and 2 mM L-glutamine. Cells were counted, seeded, and cultivated in tissue culture plates 12 h for attachment before the experimentation.

### Cellular Infection

RAW264.7 macrophages and BMDM were seeded in 24-well tissue culture plates (0.2 × 10^6^/well) and incubated overnight in CO_2_ incubator at 37°C. On the following day, cultured macrophages were infected with cultures of H37Rv. Bacterial clumps were removed by passing culture through needle 15–20 times. After 3 h of infection, macrophages were washed thrice with PBS and then cultured in gentamycin (10 μg/ml) containing medium to remove extracellular bacteria. Cells were incubated further, and bacterial growth was analyzed by plate-based method. Cell culture supernatants were collected for NO quantification. To demonstrate bacterial killing by macrophages, bacterial counts were performed over a time period of 1, 4, and 7 days. For this purpose, cell lysates were serially diluted in sterile PBS, and different dilutions were plated over Middlebrook 7H10 agar plates supplemented with OADC and Tween80. The bacterial colonies were counted and documented as colony-forming units (CFU) per milliliter.

### Quantification of Nitrate by Griess Reagent Assay

Nitric oxide production in macrophage culture supernatants in various experiments was quantified as nitrate by standard Griess reagent method. Equal volumes of the culture supernatants and Griess reagent [1% sulphanilamide/0.1% N-(naphthyl) ethylene-diaminedihydrochloride prepared in 5% o-phosphoric acid] were mixed and incubated. Absorbance was recorded at 550 nm by TECAN multimode spectrophotometer. NO titers in samples were quantified against a NaNO_2_ standard curve generated using software provided with the TECAN multimode spectrophotometer.

### Western Analysis

RAW264.7 macrophage cells or CD11b+ primary macrophages from mice were lysed in RIPA buffer (50 mM Tris-HCl, pH 7.4, 150 mM NaCl, 2 mM EDTA, 1% Nonidet P-40, and protease inhibitor mixture) and sonicated. The lysates were centrifuged at 4,000 g for 20 min at 4°C to separate the particulate fraction. Protein in cell lysates was quantified by the Bradford assay using multimode plate reader (TECAN). Cell lysates containing 25 μg of protein were dissolved with an equal volume of 2 × Laemmli buffer (Sigma), heated to 95°C for 5 min, and resolved by standard SDS-polyacrylamide gel electrophoresis (Bio-Rad) and blotted on PVDF membranes. Blots were blocked at room temperature for 1 h with 5% non-fat dry milk in TBS-T (20 mM Tris base, 137 mM NaCl, and 0.05% Tween 20, pH 7.5) and incubated overnight at 4°C with primary and subsequently with horseradish peroxidase–conjugated secondary antibodies. Blots were developed by using ECL (Millipore) reagent and normalized against β-actin.

### Preparation of Lung Homogenates for Cytokine Analysis

Lungs were weighed, placed in 1 ml of PBS solution in sterile tubes, chopped with sterile scissors into small pieces, and homogenized. Tissue homogenates were centrifuged at 4,000 g, and the supernatants were filtered through 0.22-μm filter. Filtrates were stored at −80°C until analyzed by ELISA.

### Cytokine Quantification

Cytokines were quantified using sandwich ELISA kit (R&D System, Darmstadt, Germany), and concentration of each cytokine was quantified using standard curve according to manufacturer's instructions.

### Histopathology

Mice under *M. tuberculosis* infection were sacrificed by cervical dislocation, and their lungs were excised aseptically. The lungs were perfused and fixed in PFA for H &E stating. For immunostaining, lungs were snap frozen in liquid nitrogen. Cryosections of 5 μm were generated by using Leica Cryotome, fixed in 4% PFA, and permeabilized with acetone. The lung sections were blocked with blocking buffer (PBS + 0.5% BSA + 1% chicken serum) stained with respective primary antibodies at 1:50 dilution for 1 h at room temperature, washed twice in PBS, and subsequently stained with respective Alexa Fluor 488– and 569–conjugated secondary antibodies (1:200) for 1 h. The sections were also counterstained with DAPI (1:5,000) for 10 min. Tissue sections were washed, mounted, and imaged using confocal microscope (Carl Zeiss with Zen2 version 2.1.1) at 40× magnifications with 0.65 objective numerical aperture. Fluorescent intensity of CD11b, S-1PR1, and S-1PR3 from 10 different fields of view was quantified using ImageJ software.

### Statistical Analysis

All results were expressed as the mean ± SEM of three independent experiments performed in triplicates. Statistical analysis was conducted using two-tailed unpaired *t*-test for two data sets and two-way ANOVA followed by Bonferroni posttest was used for multiple group comparisons (^*^P < 0.05; ^**^P < 0.01; ^***^P < 0.001). All of the statistical analyses were carried out with GraphPad Prism Version 5.0 software.

## Data Availability Statement

The raw data supporting the conclusions of this article will be made available by the authors, without undue reservation, to any qualified researcher.

## Ethics Statement

All animal experiments were performed as per the guidelines laid down by institutional Animal ethical committee under CPCSEA guidelines and approved by Institutional Animal ethical committee from University of Hyderabad (UH/IAEC/HP/2015/P19), Hyderabad and JALMA, Agra.

## Author Contributions

HP conceived the idea and supervised the entire study. LS, PG, PK, and SQ conducted the experiments. LS, VN, PK, and HP designed the experiments and analyzed the data. VN and LS prepared the figures. ST and SP contributed to research reagents. UG contributed in the aerosol infection of mouse. VN and HP wrote the manuscript.

### Conflict of Interest

The authors declare that the research was conducted in the absence of any commercial or financial relationships that could be construed as a potential conflict of interest.
