# Supplementary material for: Sphingosine-1-Phosphate (S-1P) Promotes Differentiation of Naive Macrophages and Enhances Protective Immunity Against Mycobacterium tuberculosis
Source: Front Immunol. 2020 Jan 24;10:3085. doi: 10.3389/fimmu.2019.03085 (PMC6993045; doi:10.3389/fimmu.2019.03085)
Supplement: Supplementary file 1 [file Data_Sheet_1.pdf]

Supplementary Material

Suppl Fig. 1

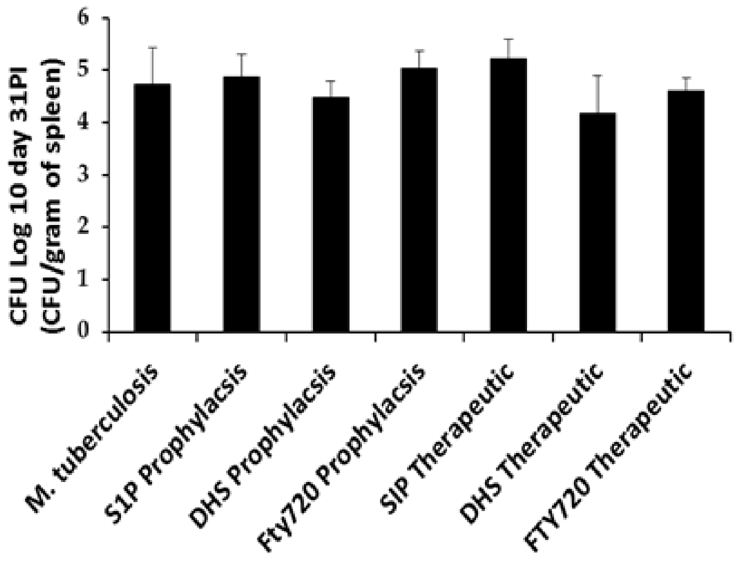

Suppl. Figure1. Spleens from mice challenged with M. tb in Figure 3A were excised and the numbers of bacterial colonies were analyzed using plate-based method. Shown here is CFU ± SEM from several mice used in various indicated groups.

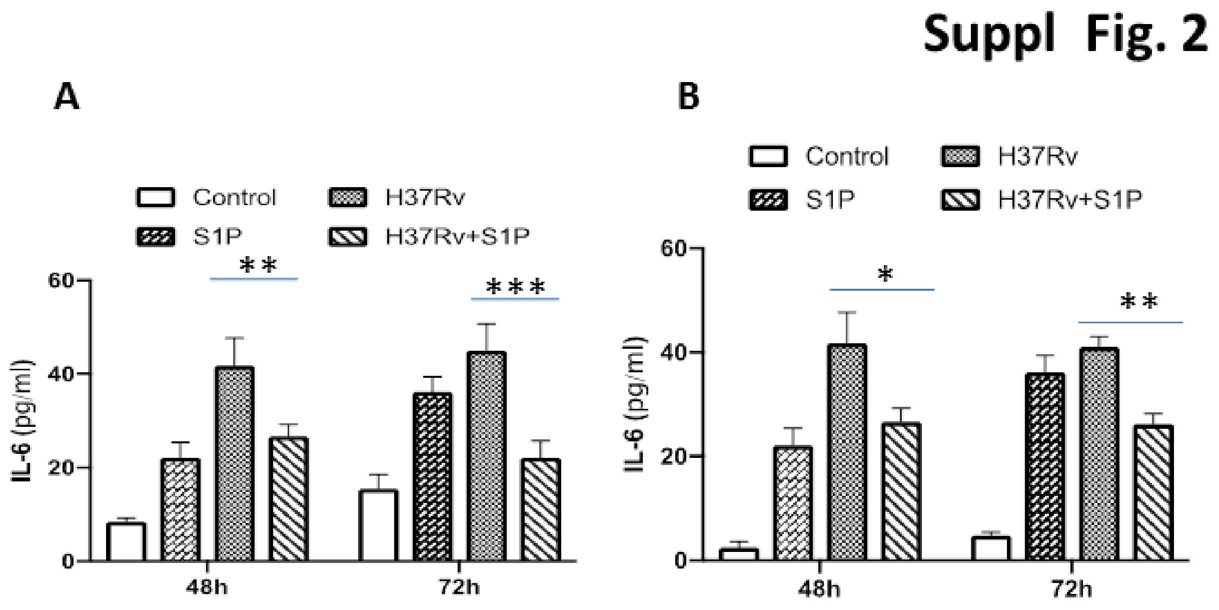

Suppl. Figure 2. RAW 264.7 macrophages and BMDM were infected with *M. tb* in presence of S-1P and IL-6 titre was quantified in their culture supernatants. Shown here is the pg/ml of IL-6 ± SEM released during the course of infection from three independent experiment. Statistical analysis were conducted using two-way Anova followed by Bonferroni post-test (*P<0.05, **P<0.01, ***P<0.001

Suppl Fig. 3

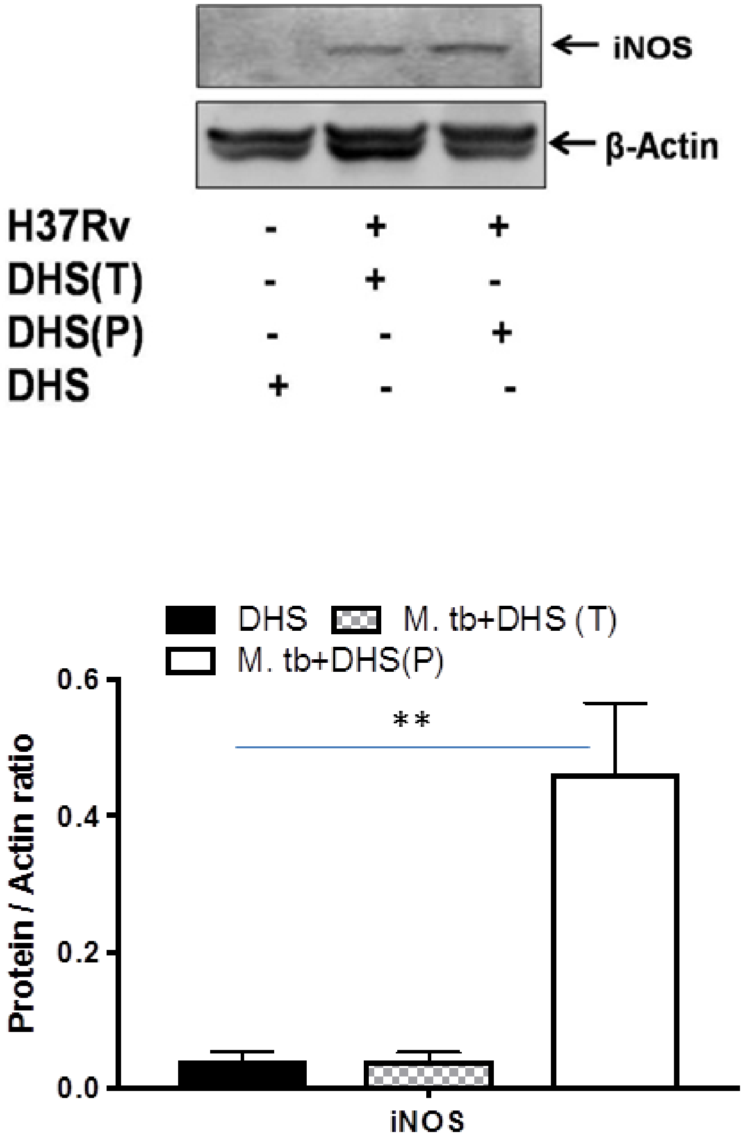

Suppl. Figure 3. Whole lung lysates from DHS treated mice were purified and analysed for M1 effector protein (iNOS) by western blot. Shown here are the representative blots from each groups. β-actin was used as a loading control. Densitometric analysis of the blot shown was quantified by ImageJ software and the values were plotted in terms of relative protein expression. Statistical analysis were conducted using two-way Anova followed by Bonferroni post-test (*P<0.05, **P<0.01, ***P<0.001).

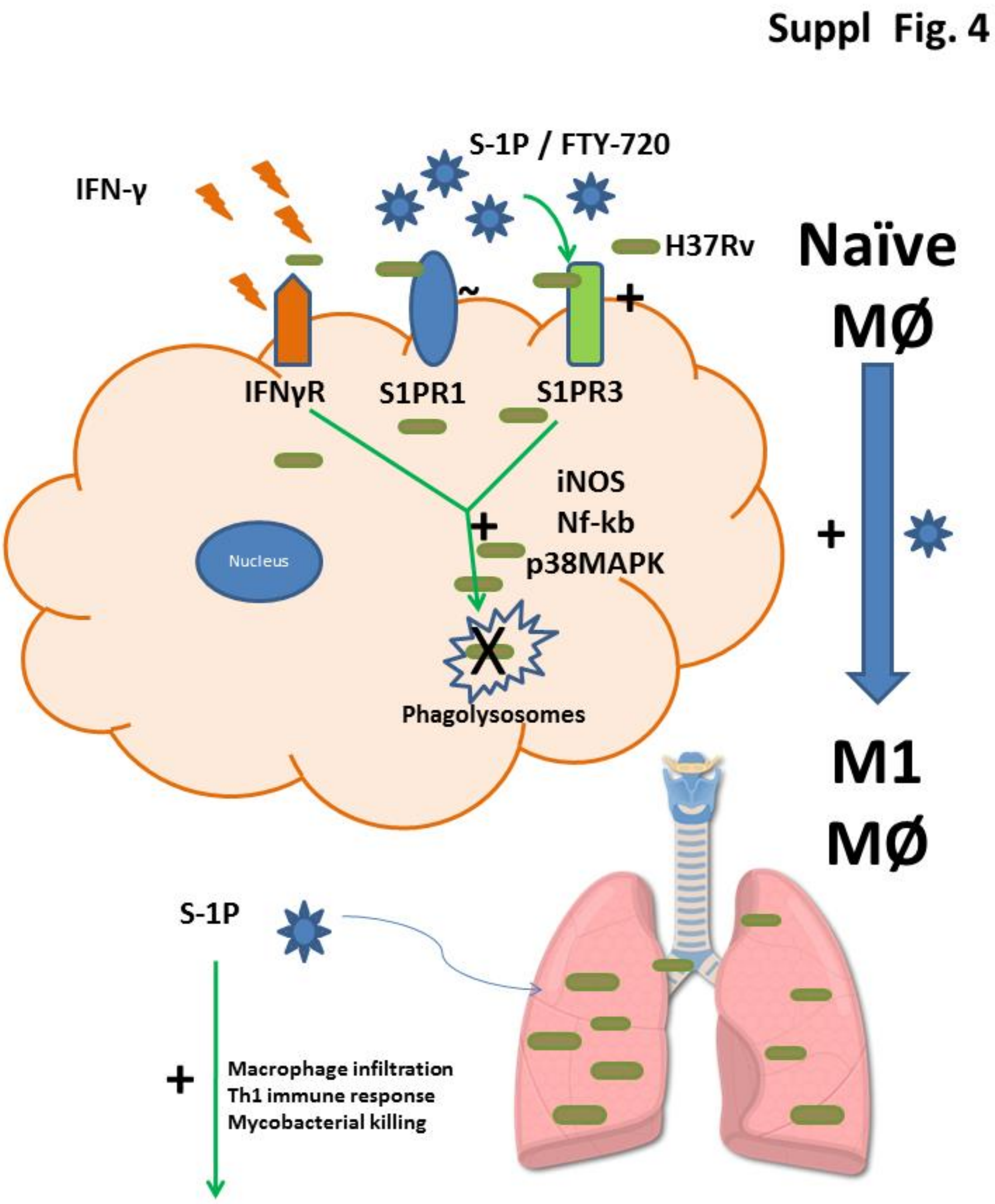

Suppl. Figure4. Schematic representation of possible intra as well as extracellular S-1P receptor signalling components in macrophages and lung tissue for controlling infection, with special focus on S-1PR2 and S-1PR3 signaling for optimum defence against pathogenic mycobacteria.
